# Preparation and use of nanogels as carriers of drugs

**DOI:** 10.1080/10717544.2021.1955042

**Published:** 2021-07-26

**Authors:** Cuixia Li, Sreekanth Reddy Obireddy, Wing-Fu Lai

**Affiliations:** a Key Laboratory of Analytical Science and Technology of Hebei Province, College of Chemistry & Environmental Science, Hebei University, Baoding, China; b Department of Chemistry, Sri Krishnadevaraya University, Anantapur, India; c School of Education, University of Bristol, Bristol, UK; d Ciechanover Institute of Precision and Regenerative Medicine, The Chinese University of Hong Kong (Shenzhen), Shenzhen, China

**Keywords:** Nanogels, synthesis, carriers, drug delivery, sustained release

## Abstract

Nanogels have high tunability and stability while being able to sense and respond to external stimuli by showing changes in the gel volume, water content, colloidal stability, mechanical strength, and other physical/chemical properties. In this article, advances in the preparation of nanogels will be reviewed. The application potential of nanogels in drug delivery will also be highlighted. It is the objective of this article to present a snapshot of the recent knowledge of nanogel preparation and application for future research in drug delivery.

## 1. Introduction

Nanomaterials can enable the controlled release of drugs and improve the stability of those agents, making a new way to disease treatment (Lang et al., 2020). Hydrogels are three-dimensional networks of polymers containing hydrophilic groups which are cross-linked by covalent bonds, hydrogen bonds, van der Waals forces or various other interactions. They can absorb and retain a substantial amount of fluids without undergoing dissolution. This unique property enables hydrogels to be used in tissue engineering and drug delivery (Peppas et al., 2006; Brandl et al., 2007; Bawa et al., 2009;; Das et al., 2021; Nai et al., 2021). Nanogels are hydrogels with the size being around 1–1000 nm (Ahmed et al., 2020). They can carry and protect the active ingredients loaded inside, and can sense and respond to external stimuli by exhibiting changes in the gel volume, water content, colloidal stability, mechanical strength, and other physical/chemical properties (Wu & Tian, 2008; He et al., 2009). Along with their high tunability and stability (Alarcón et al., 2005; Sahiner et al., 2006; Lu et al., 2021), nanogels have been used extensively in drug delivery.

Similar to biological tissues, nanogels have good biocompatibility. Meanwhile, the 3D structure enables hydrophobic or hydrophilic drugs to be encapsulated inside. This protects the drugs from degradation during storage or blood circulation (e.g. hydrolysis or enzymatic degradation) and reduces toxic side effects (Raemdonck et al., 2009; Pan et al., 2012; Maciel et al., 2013; Chen et al., 2017; Liu & Thayumanavan, 2017). Importantly, nanogels can be loaded with drugs with the drug activity retained (Vinogradov, 2010; Peng et al., 2021). In addition, the blood circulation time as well as tissue targeting capacity of nanogels can be prolonged by surface modification (Vinogradov et al., 2002). This further enhances the performance of nanogels in pharmaceutical formulation. Nanogels, therefore, have attracted the attention of many researchers.

## 2. Methods of nanogel preparation

Over the years, a variety of methods for the synthesis of nanogels have been developed (Nicolas & Jutta, 2010; Jiang et al., 2014; Torchilin, 2014). Depending on the raw materials adopted, nanogels can be synthesized by either having polymerization and crosslinking being held simultaneously or having polymerization being performed first followed by crosslinking.

### 2.1. Concomitant polymerization and crosslinking

Nanogels can be synthesized by having polymerization and crosslinking being held concomitantly. Since most of the monomers and crosslinking agents used for nanogel preparation are water-soluble, polymerization reactions are generally carried out in an aqueous medium. Based on the working mechanism, methods of nanogel preparation in which polymerization and crosslinking are held concomitantly can be divided into three types: precipitation polymerization, inversion emulsion polymerization and micro-template polymerization.

#### 2.1.1. Precipitation polymerization

A major feature of precipitation polymerization is that the reaction system is homogeneous. In another word, all monomers, crosslinkers and initiators before the reaction are homogeneously dissolved in the same reaction medium. The length of the polymer chain increases as the polymerization reaction progresses. When the polymer chain grows to a certain length, the generated phase is separated out to form polymer colloidal particles and finally nanogels. The number of monomers, crosslinking agents and initiators all have an effect on the size of nanogels.

Precipitation polymerization is one of the first methods exploited for the generation of temperature-responsive poly(N-isopropylacrylamide) (PNIPAM) nanogels (Oh et al., 2008; Raemdonck et al., 2009). During nanogel preparation, the insertion of the degradable crosslinker enables the nanogels to have controlled drug release in tumor microenvironments (Zhang et al., 2015; Wang et al., 2016; Zhang & Tung, 2018; Liu et al., 2019; Liu et al., 2020). For example, nanogels prepared from crosslinking agents containing disulfide bonds enable accelerated drug release triggered by the high concentration of glutathione in tumor cells (Raemdonck et al., 2009; Pan et al., 2012; Maciel et al., 2013; Chen et al., 2017). Nanogels containing acetals and ketals can release the loaded drug rapidly in the tumor acidic environment (Chen et al., 2007; Liu & Thayumanavan, 2017). Chen et al. (2007) synthesized PNIPAM nanogels by using the precipitation polymerization method and tested the particle size distribution of the nanogels with a transmission electron microscope and Malvin particle size analyzer. They found that the particle size of the nanogels could be adjusted by changing the content of the surfactant sodium dodecyl sulfate. Duracher and cowrokers (Zhang et al., 2004; Ribovski et al., 2021) studied the mechanism of copolymerization reactions between N-isopropylacrylamide (NIPAM) and various other hydrophilic monomers to form PNIPAM nanogels. The aqueous solution of PNIPAM has a minimum critical solution temperature (LCST) of about 32 °C. The reaction temperature must be higher than the LCST and is thus set at 60–80 °C. When the resulting PNIPAM free radical chain grows to a certain critical chain length, the originally hydrophilic chain changes to a hydrophobic chain, resulting in ‘coil-to-globule’ conformational transformation, with the primary particles appearing in the reaction system. This is the nucleation stage of the precipitation polymerization reaction.

Ribovski et al. (2021) used precipitation polymerization to prepare fluorescently labeled PNIPAM nanogels. The hardness of PNIPAM nanogels is regulated by the degree of polymer crosslinking. The effect of the hardness of PNIPAM nanogels on their interactions with the blood-brain barrier (BBB) model in vitro was studied. PNIPAM nanogels of ∼200 nm with varying degrees of stiffness were made by inclusion of 1.5 mol% (NG1.5), 5 mol% (NG5), and 14 mol% (NG14) of N,N′-methylenebis(acrylamide) (BIS) during synthesis. NG14 nanogels showed a higher level of uptake by brain endothelial cells than NG1.5 and NG5 nanogels. In addition, NG1.5 and NG5 exhibited a higher level of transcytosis compared to NG14. An increase in the size of the nanogels (up to ∼400 nm) without changing the nanogel stiffness was shown to have little influence on cellular uptake or transcytosis. All these reveal that the hardness of nanogels has an opposite effect on the uptake and endocytosis of nanogels in the BBB.

In precipitation polymerization, various colloidal particles can be used as templates or seeds, and the stimuli-responsive polymer can be used to coat the template particles to form multifunctional or stimuli-responsive composite nanogels (Zha et al., 2002). Zha et al. (2002) used the precipitation polymerization method to coat the surface-modified silica colloidal particles with PNIPAM, and then removed the silica particles to obtain the temperature-responsive nanogels with a hollow structure. The size of the hollow nanogels can be controlled by changing the size of the template. The thickness of the hollow nanogel shell can also be adjusted by changing the mass ratio of the monomer and the template. The volume phase transformation of the hollow nanogels occurs at about 32 °C. The permeability of hollow nanogel shells can be changed in response to external stimuli, and this mediates the controlled release of guest molecules. Recently, Xing et al. (2011) also adopted a similar method to synthesize pH/temperature-responsive hollow nanogels. By changing the temperature, the hollow nanogels can be loaded with drugs, with the rate of drug release being able to be controlled by changing the pH of the surrounding medium (Averick et al., 2011).

#### 2.1.2. Inverse emulsion polymerization

**Figure 1.**
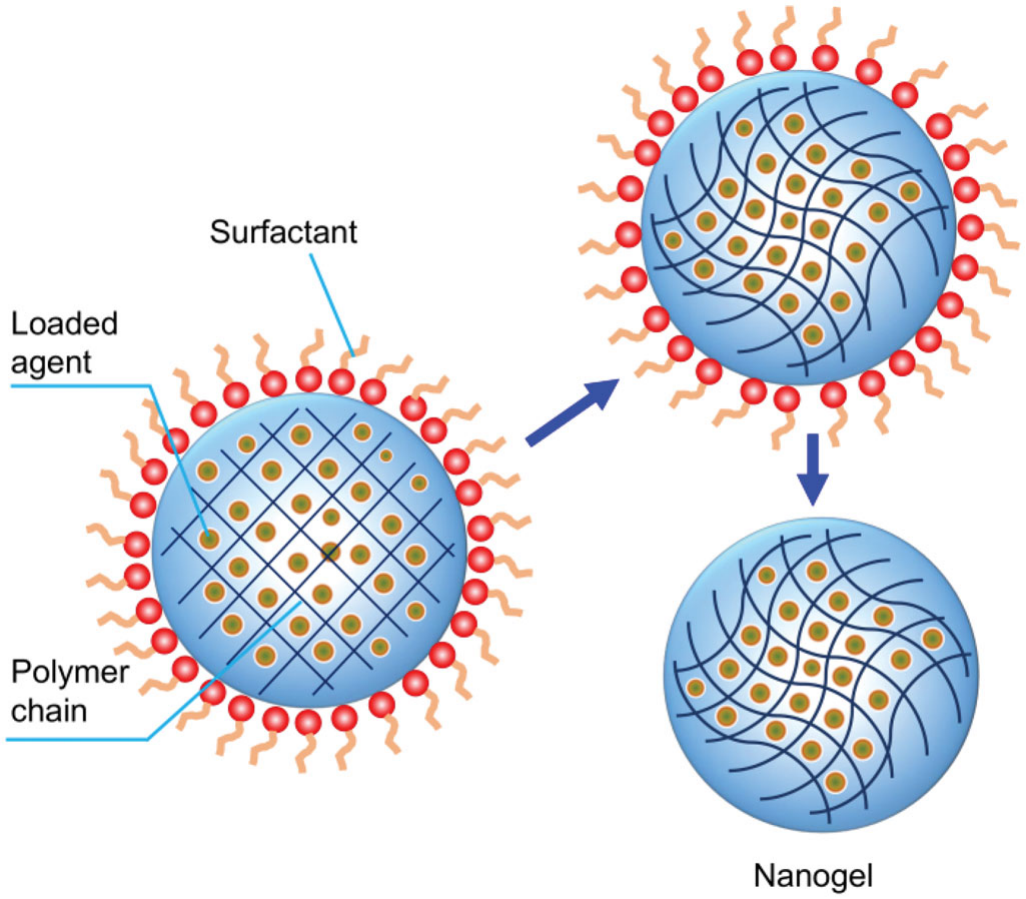
Generation of nanogels via the inverse emulsion polymerization method.

Synthesis of nanogels can occur in the presence of an oil-soluble emulsifier upon the adoption of proper emulsification methods. The generated W/O inverse emulsions can undergo polymerization reactions. After removing both the organic solvent and the emulsifier, nanogels that can be stably dispersed in an aqueous medium can be obtained (Figure 1) (Wang et al., 2018). During inverse emulsion polymerization, the size of nanogels is affected by many factors, including the choice of surfactants, the concentration of the monomer and the crosslinker, and the pH of the reaction medium (Mitra et al., 2001; Peres et al., 2018). The disadvantage of this method is that an organic solvent is used as the reaction medium. The presence of emulsifiers or co-emulsifiers will bring difficulties to the purification of the generated nanogels.

**Figure 2.**
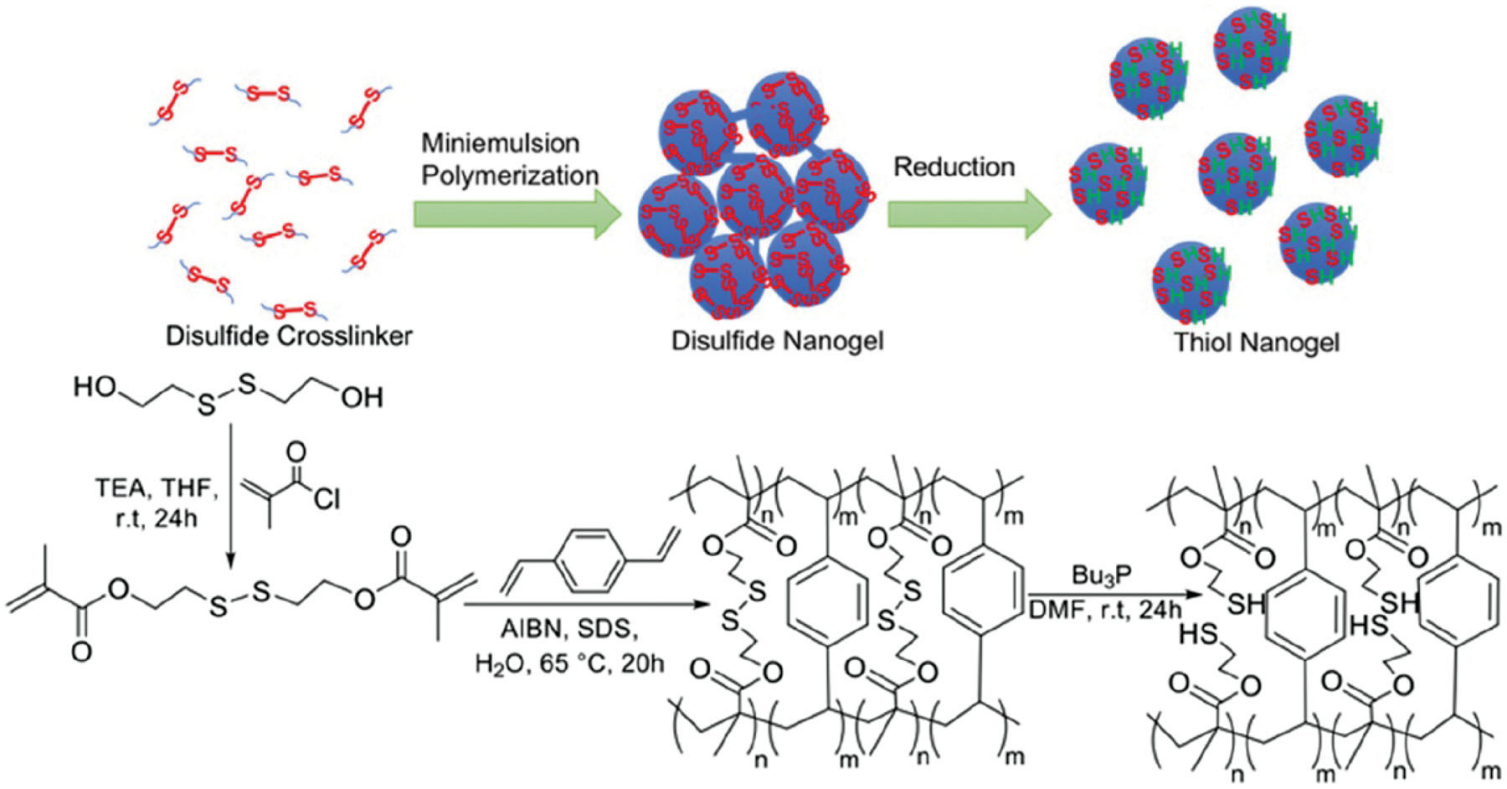
A synthetic method for the generation of thiol polymer nanogels. AIBN: 2,2′-azobis(2-methylpropionitrile); Bu_3_P: tributylphosphine: SDS: sodium dodecyl sulfate; TEA: trimethylamine; THF: tetrahydrofuran; r.t.: room temperature; DMF: *N*,*N*-dimethylformamide. Reproduced from Zhang et al. (2021) with permission from the American Chemical Society.

Using N, N′-methylenebis(acrylamide) (BIS) and N-acryloyl-l-glutamic acid (l-AGA), Peres et al. (2018) prepared poly(l-AGA) and poly(l-AGA-co-Bis) nanogels by inverse emulsion polymerization. The results showed that the degree of nanogel swelling increases with a change of pH. In addition, the presence of carboxylic acid groups and amide groups in the polymer network plays an important role in determining the physicochemical properties of the nanogels and in affecting the overall hydrophilicity. Deen et al. (2008) copolymerized N-acryl-N'-methylpiperazine with methyl methacrylate in an O/W reverse-phase microemulsion system to prepare pH-responsive nanogels. Nanogels swell in an acidic aqueous solution but shrink in an alkaline solution. Zhang et al. (2021) adopted the microemulsion polymerization method and introduced a high density of thiol groups into the polymer gel to obtain disulfide polymer nanogels. After forming the nanogels, the disulfide and tributyl phosphine (Bu_3_P) were reduced to mercaptan groups to obtain the thiol polymer nanogels. The combination of the hydrophobic benzene ring and the hydrophilic sulfhydryl group in the nanogel repeat unit makes the nanogel amphiphilic (Figure 2). The nanogel has a high sorption affinity for Hg(II) complexes and Hg-dissolved organic matter complexes found in water and for elemental (Hg0) and soluble Hg-alkyl thiol species found in hydrocarbons.

Matyjaszewski and coworkers (Zhang et al., 2021) synthesized poly(ethylene glycol) methacrylate monomethyl ether ester (OEOMA) nanogels using atomic transfer radical polymerization (ATRP) in a microemulsion system with the disulfide serving as the crosslinking point. Compared with conventional reversed microemulsion polymerization, the nanogels synthesized by using active free radicals have higher colloidal stability, narrower and more uniform distribution of size, and a more controllable structure and composition. A large number of active groups on the surface of the synthesized nanogels can be used to be conjugated with other active components (Dai et al., 2006). If this method is used to synthesize nanogels with degradable crosslinkers, the molecular weight distribution of the nanogels can be narrower, and the nanogels can be more easily metabolized in a human body. Recently, Peng et al. (2021) also designed a degradable poly(phosphorylcholine)-based (^H^PMPC) nanogel for drug release induced by hypoxia in tumor cells. The obtained nanogel showed a prolonged blood circulation time and led to a negligible immune response. Importantly, the nanogel was found to be effectively degraded. Drug-loaded ^H^PMPC nanogels, therefore, showed promising tumor inhibition effects both in vivo and in vitro while having high biocompatibility.

Monodisperse nanogels can be prepared by using the membrane emulsification technology (Seiffert and Weitz, 2010). However, being limited by the pore size of the emulsion membrane, it is difficult to prepare nanogels with a particle size less than 100 nm by using this method. Microfluidic emulsification involves mixing and emulsifying the aqueous solutions containing the monomer, crosslinker, and initiator through microchannels in the presence of an organic phase to form O/W emulsions, which are then polymerized in situ to form nanogels. The shape and size of the nanogels are affected by the size of microchannels, the flow rate of the solutions and the polymerization reaction time (Oudshoorn et al., 2008).

#### 2.1.3. Microtemplate polymerization

During microtemplate polymerization, the monomer and the crosslinking agent are first added to the microtemplate. They then undergo free radical polymerization. Finally, the hydrogel nanoparticles are separated from the microtemplate. The advantage of this method is that it can generate nanogels with different shapes (Grimaudo et al., 2019). In addition, the method allows cells or other bioactive components to be easily loaded into the nanogels; however, due to the limitation of the size of the microtemplate, it is difficult to prepare nanogels with a relatively small size. One type of microtemplate polymerization is the photolithographic microtemplate polymerization method, in which the monomer and the crosslinking agent are mixed with the photoinitiator. Upon coating on a non-wetting substrate, a layer of a non-wetting template with micro-holes is added. After illumination with a light source with a specific wavelength, free radical polymerization of the monomer and the crosslinking agent is triggered.

The most prominent feature of lithographic microtemplate polymerization is that it can precisely control the size, shape and composition of the generated nanogels. Nanogels prepared using this method can have different shapes with a particle size less than 200 nm. Yet, this method has stringent requirements on the surface of the template and the substrate. Polymethylsiloxane and photocurable perfluorinated polyether are some of the possible templates and substrate materials (Oudshoorn et al., 2007). Grooved microtemplate polymerization is similar to photolithographic microtemplate polymerization. During nanogel preparation, the monomer and the crosslinking agent are loaded into the microtemplate with grooves. The hydrogel particles with a fixed size and shape are then polymerized by light irradiation (Yeh et al., 2006; Jung et al., 2009).

### 2.2. Separate polymerization and crosslinking

In this category, polymers are first formed, followed by crosslinking between the polymer molecular chains to generate nanogels. This method is especially suitable for the preparation of nanogels based on natural polymers (Xia et al., 2003). Based on the mechanism of nanogel generation, the method can be of different types: precipitation/crosslinking, emulsification/crosslinking, self-assembly/crosslinking, and microtemplate forming/crosslinking.

#### 2.2.1. Precipitation/crosslinking

The precipitation/crosslinking method is to have water-soluble polymers being precipitated out of a homogeneous aqueous solution to form nanoparticles. After that, crosslinking reactions are performed to make the polymers in the particles crosslinked to generate nanogels. Xia et al. (2003) prepared nanogels in a salt solution without using an emulsifier. The size and size distribution of the nanogels are closely related to the salt concentration and the reaction temperature. The LCST of the aqueous solution of the polymer decreased with the increase of the NaCl concentration. Then, crosslinking reactions are performed. Unfortunately, the functioning of the crosslinking agent used to prepare the nanogels requires the availability of a strongly alkaline medium, which in general is not available during the drug loading process. For this, Cai et al. (2003) first modified the molecular chain of the polymer so that its side chain was either connected to the vinyl group through the degradable ester linkage or directly connected to the acrylate group. Then the free radical polymerization reaction was initiated by heating the polymer solution above the LCST. Recently, Shen et al. (2007) prepared pH-responsive nanogels with the particle size of 70-80 nm by adding an ethylenediamine diacetic acid diacetaldehyde (EDTAA) crosslinking agent into an aqueous solution of chitosan. The nanogel has a positive charge when pH <4.8, and a negative charge when pH > 5.2. The change of pH of the surrounding medium can make the surface charge of the nanogel reverse. Nanogels can also be formed through electrostatic interactions if some polyelectrolytes with specific structures are added into an aqueous solution of chitosan (Ethirajan et al., 2008). The concentration of water-soluble polymers used to prepare nanogels by using this method should not be too high; otherwise, large gels are easily formed. The efficiency of this preparation method is, therefore, relatively low.

#### 2.2.2. Emulsion/crosslinking

**Figure 3.**
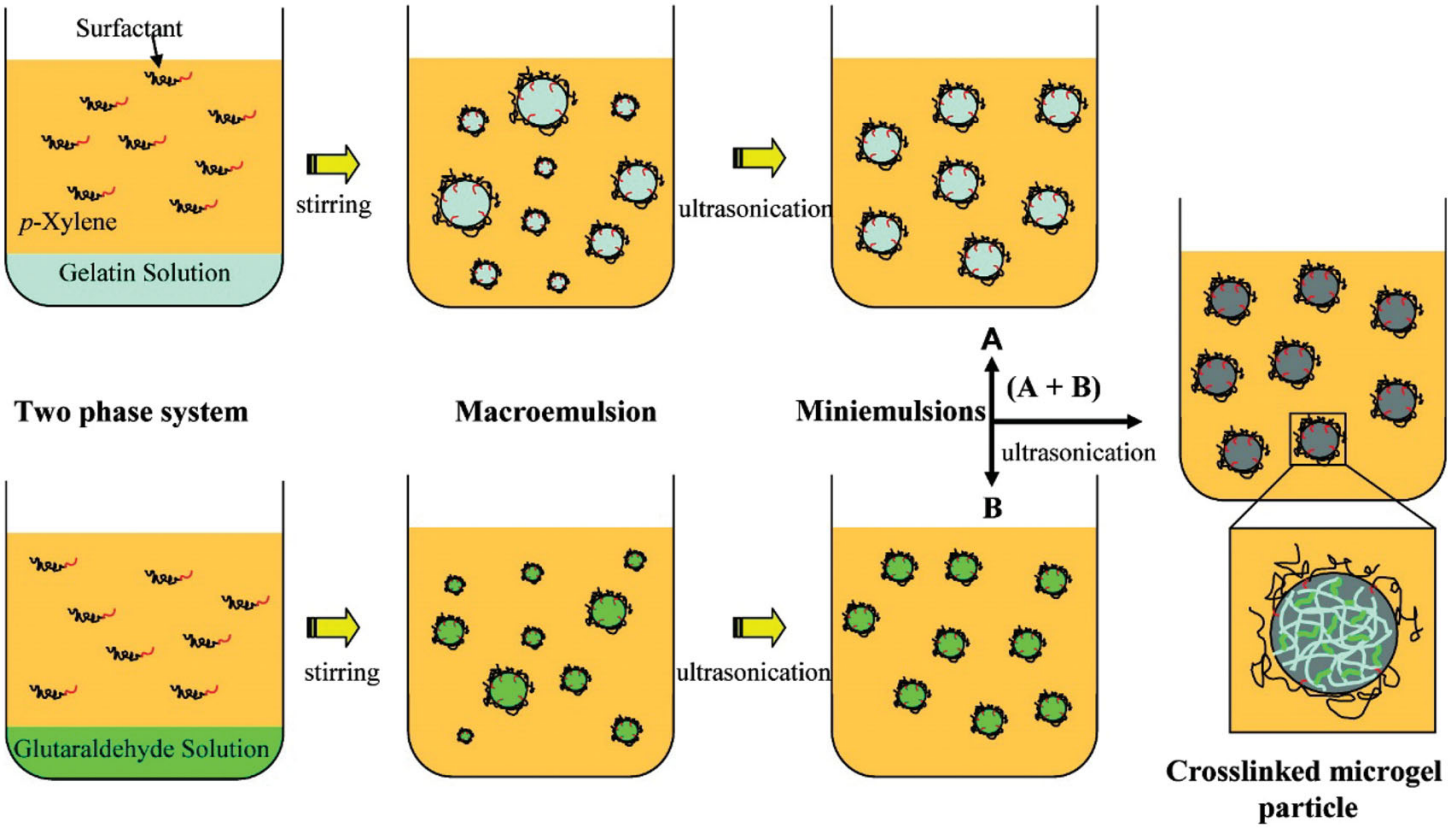
A schematic illustration depicting the generation of nanogels using the emulsification/crosslinking method. Reproduced from Ethirajan et al. (2008) with permission from the American Chemical Society.

In this method, polymers are first dissolved in water. Under the action of oil-soluble surfactants, appropriate emulsion methods are applied to form an aqueous dispersion of polymers in an organic solvent to obtain W/O inverse emulsions in the presence of a surfactant. As shown in Figure 3, an aqueous solution of gelatin was emulsified into a fine emulsion under the action of an ultrasonic wave. Then glutaraldehyde was added as a crosslinking agent to generate nanogels (Mitra et al., 2001). Mitra et al. (2001) dispersed the aqueous solution of dextran-doxorubicin (DOX) conjugate and chitosan in organic solvents to form microemulsions. After adding a crosslinking agent, pH-responsive nanogels that can be used to deliver anti-cancer drugs were prepared. The crosslinking reaction between polymers occurs in tiny droplets of an aqueous solution under mild reaction conditions. Therefore, it can be used to generate nanogels loaded with fragile bioactive agents. Similar to inversion emulsion polymerization, this method involves the use of organic solvents, emulsifiers and co-emulsifiers. This brings great difficulties to the purification of nanogels.

The membrane emulsification method can generate a W/O invert emulsion from an aqueous solution of a polymer. The emulsion can then undergo crosslinking reactions to generate monodisperse nanogels (Wang et al., 2006). Due to the high viscosity of the polymer solution and the limitation of the pore size, this method can hardly prepare nanogels with a relatively small size. It is, however, convenient for drug loading (Zhang et al., 2007). The microfluidic emulsification technology can also be used to prepare nanogels. This was shown by Zhang et al. (2007), who used the microfluidic emulsification method to generate nanogels from alginate through crosslinking mediated by using Ca^2+^. The generated nanogels are stable, with the morphology and structure being easy to be controlled.

**Figure 4.**
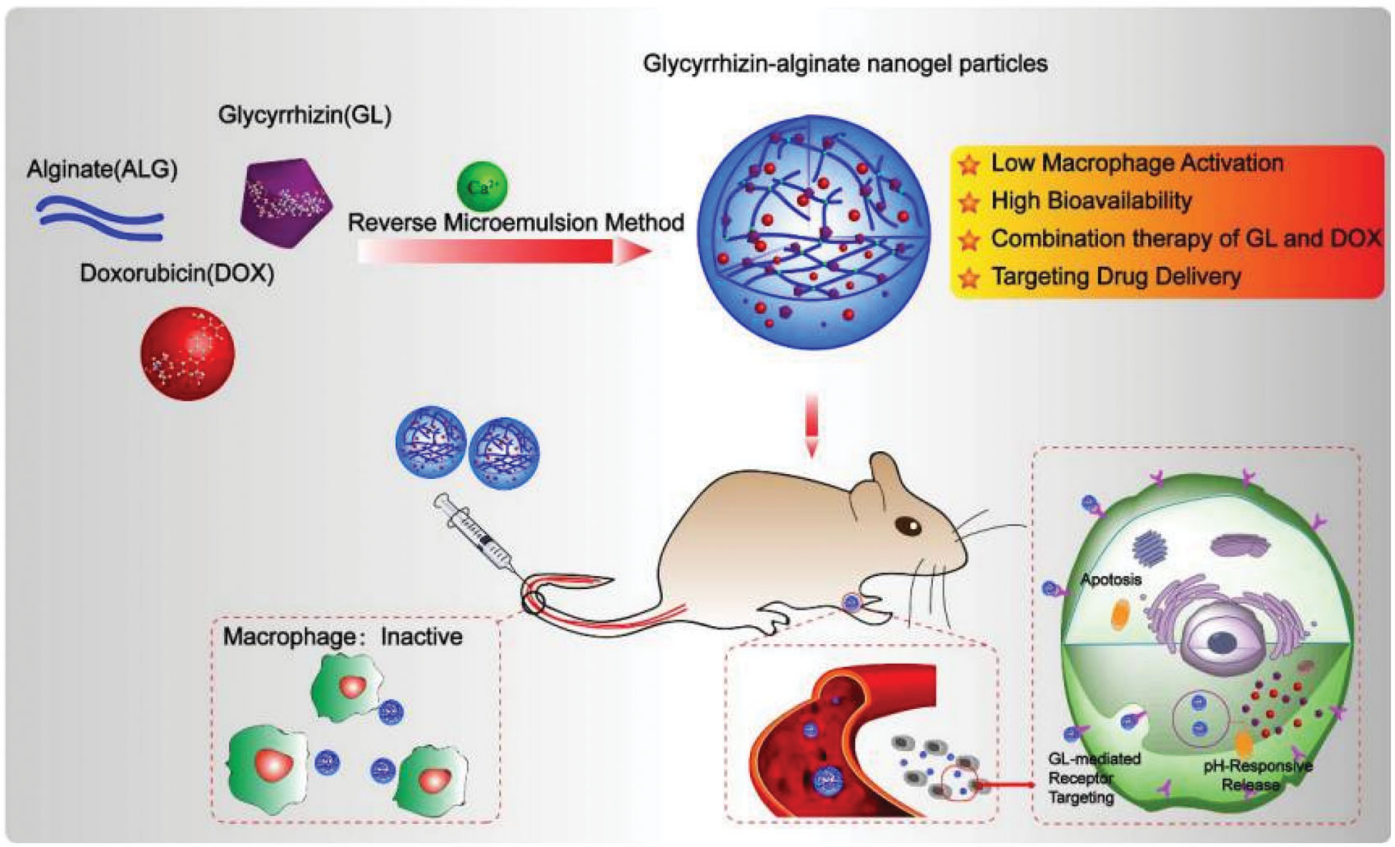
A schematic illustration depicting the generation and use of alginate-based nanogels co-loaded with glycyrrhizin and doxorubicin (DOX/GL-ALG NGPs) for cancer therapy. Reproduced from Wang et al. (2019) with permission from Ivyspring International Publisher.

Nanogels provide a platform for drug co-delivery because they have a 3D network structure for being loaded with both hydrophilic and hydrophobic compounds. In a recent study, both glycyrrhizic acid (GL) and doxorubicin (DOX) were loaded into alginate-based nanogels by using the phase-transition temperature emulsification method. The obtained drug-loaded alginate-based nanogels exhibited not only the hepatocellular carcinoma targeting property but also the synergistic antitumor effects mediated by GL and DOX (Figure 4) (Tong et al., 2018). In addition, the alginate-based nanogels showed good biocompatibility and low toxicity to liver tissues and enabled controlled release of the drugs (Sahu et al., 2017). All these suggest that nanogels have the practical potential for use in multi-drug therapy.

Apart from the aforementioned, the solvent-evaporation emulsification method can be used to generate nanogels (Bagheri et al., 2021). Bagheri et al. (2021) generated nanogels from chitosan-carboxymethyl cellulose and flaxseed oil and then loaded the nanogels with atorvastatin to obtain atorvastatin-oil nanogels (ATONG). The loading efficiency, drug release sustainability, and gel stability of ATONG were tested. The results confirmed the high efficiency of drug loading and release. Cytotoxicity tests showed that ATONG can safely release atorvastatin upon cellular uptake. Recently, flaxseed oil-atorvastatin nanogels were successfully generated from chitosan, carboxymethyl cellulose and poly(vinyl alcohol). The obtained nanogels exhibited negligible toxicity and antibacterial properties.

#### 2.2.3. Self-assembly/crosslinking

Molecular self-assembly refers to the process in which molecules spontaneously form thermodynamically stable aggregates through non-covalent interactions. Driving forces of molecular self-assembly include hydrogen bonding interactions, electrostatic interactions, hydrophobic interactions, and van der Waals forces. Water-soluble polymers with specific structures can self-assemble into nanogels driven by these interactions (Meng et al., 2009). Physical crosslinks in nanogels are not stable. Dissociation is easy to occur under high temperature or high salt concentrations. The stability of self-assembled nanogels can be significantly improved if chemical crosslinking and optical crosslinking are used instead. If disulfide bonds are involved in crosslinking, the prepared nanogels can respond to the degradation of specific reducing substances such as glutathione in cells (Sawada & Akiyoshi, 2010). The conditions for self-assembly/crosslinking during the generation of nanogels are relatively mild, so this method can be used to load the nanogels with fragile bioactive components such as proteins (Dou & Jiang, 2007). The size of the assembled nanogels can be controlled by selecting an appropriate polymer concentration or environmental parameters such as temperature, pH and ionic strength.

In fact, the self-assembly/crosslinking method is particularly suitable for the preparation of nanogels based on natural polymers (Xia et al., 2003; Rolland et al., 2005; Napier & Desimone, 2007). There are many hydroxyl groups on the molecular chains of natural polysaccharides, which can be grafted with and modified by polymers containing carboxyl groups. The copolymers generated can self-assemble through hydrogen bonding interactions, and upon further crosslinking reactions, nanogels can be obtained. Dou & Jiang (2007) grafted modified hydroxyethyl cellulose (HEC) with poly(acrylic acid) (PAA), and the generated copolymer (HEC-g-PAA) could self-assemble into nanoparticles in an aqueous medium. The molecular chains of PAA could be crosslinked with a crosslinking agent to finally generate pH-responsive nanogels.

#### 2.2.4. Microtemplating/crosslinking

The method is to add precursors of chemical reactions into a microtemplate and then obtain nanogels upon chemical crosslinking or optical crosslinking. It is also called the ‘Particle Replication in Nonwetting Templates (PRINT)’ method. The method can generate monodisperse nanogels with a well-controlled size, shape and composition (Ito, 1999; Euliss et al., 2006; Gratton et al., 2008; Chen et al., 2013). Gratton et al. (2008) used this method to prepare nanogels with different sizes, shapes and properties. Results showed that rod-like nanogels with positive surface charge can undergo cellular uptake most efficiently. Ito (1999) also used the method to prepare pH-responsive nanogels from tetraazidoaniline-modified PAA.

Liposomes are hollow vesicles with a double-walled structure formed by the self-assembly of amphiphilic phospholipid molecules. Their cavities can be used as microtemplates to prepare nanogels. For example, Hong et al. (2008) took the liposome core as a template. They first coated the liposome core with sodium alginate, followed by the addition of an aqueous solution containing Ca^2+^. When the temperature of the solution exceeded the melting point of the liposome, Ca^2+^ in the solution entered the nucleus through the liposome shell to crosslink molecules of sodium alginate. After adding a surfactant to remove liposomes on the surface, nanogels with the particle size of 120–200 nm were obtained. The nanogels were found to respond to changes in the ionic strength of the surrounding medium.

## 3. Applications in drug delivery

Nanogels have been widely used as drug carriers partly because of their capacity of being loaded with agents having different properties (Table 1) (Vinogradov et al., 2002; Morimoto et al., 2006; Oh et al., 2007; Oh et al., 2008; Ashrafizadeh et al., 2019). When nanogels are developed for drug delivery purposes, monomers are selected according to the mechanism of action of the drug to be delivered (Vinogradov, 2007; Bae et al., 2008). Nanogels have good biocompatibility, high stability, and high functional flexibility. These can facilitate the use of nanogels in drug delivery (Peppas et al., 2006; Zhang et al., 2016; Vashist et al., 2018). In addition, the pores in the three-dimensional network structure of nanogels enable the inclusion of small-molecule compounds. Nanogels can control the process of drug release, shield the odor of the loaded drug, improve the therapeutic effect and reduce adverse drug reactions (Ganachaud et al., 1997; Verma & Somia, 1997; Morimoto et al., 2006). Practically, there are three ways in which non-genetic drugs are loaded into nanogels. The first one is in the form of a storage structure, in which drug molecules are concentrated in the inner layer, with the outer layer being a gel film. The second one is in the form of having the drug molecules uniformly dispersing inside the nanogel. The third one is the one in which drug molecules bind to the gel via covalent interactions. At present, research on nanogels mainly focuses on overcoming the instability of protein drugs and achieving tissue targeting. Contrary to chemical drugs, biomacromolecules such as proteins have a large molecular weight and a complex structure. Delivering them while maintaining their properties is more challenging than delivering small-molecule compounds (Li et al., 2002; Cheng et al., 2011; Thorne et al., 2011).

**Table 1.** Examples of nanogels reported for drug delivery applications.

Agent delivered	Nanogel adopted	Properties	Ref.
DOX	^H^PMPC nanogel	Showing good tumor inhibition effects and good biocompatibility	Peng et al., 2021
Water-soluble agent	pH-responsive nanogel	Showing pH-responsive swelling properties	Jung et al., 2006
Cu(II) complex	Oligo(ethylene oxide) methacrylate nanogel	Possessing a large number of active groups (which can be used for surface modification) on the nanogel surface and showing pH-responsive swelling properties	Zhang et al., 2021
GL and DOX	Alginate-based nanogel	Showing tumor-targeting capacity, good tumor inhibition effects, good biocompatibility, and high drug release controllability	Tong et al., 2018
Atorvastatin	Atorvastatin-oil nanogel	Having the capacity of permeating through skin layers	Kabanov & Vinogradov, 2009
Water-soluble agent	PNIPAM nanogel	Having good target specificity and high drug release controllability	Duracher et al., 1999
Hydrophobic and hydrophilic agents	Dual chemically and physically crosslinked anionic nanogel	Showing high efficiency in being loaded with both hydrophilic and hydrophobic agents	Bae et al., 2008
Oligodeoxynucleotide	Cationic aminated latex nanogel	Having a high affinity for the oligodeoxynucleotide for nucleic acid delivery	Ganachaud et al., 1997

When nanogels reach the target tissue, the onset and rate of drug release have to be properly controlled. There are three major methods of achieving this: (1) drug release via gel degradation, (2) drug release via gel expansion and drug diffusion, and (3) drug release via the breakage of interactions between the drug and the nanogel. In addition, a nanogel can be designed to be either a programmed pulsed-release system (PPRS) or an intelligent pulsed-release system (IPRS). The release mode of a PPRS is achieved by the predetermined gel structure, with the lag time and duration of drug release being controlled by the polymer degradation profile. Recently, Bütün et al. (2000) designed a core-shell gel-based sustained-release system, which consisted of alternating drug-containing and non-drug-containing regions, in which the duration of drug release was determined by the gel properties and by the thickness of each layer. Besides delivering non-genetic agents, nanogels can be used to deliver nucleic acids to prevent the occurrence of problems caused by the immunogenicity and pathogenicity of viral vectors (Verma & Somia, 1997). By taking advantage of the surface charge of nanogels, nucleic acids can be effectively loaded for delivery purposes. The feasibility of this was demonstrated by Ganachaud et al. (1997), who synthesized aminated core-shell particles via soap-free emulsion polymerization of styrene and vinylbenzoamide. The particles were found to be successfully loaded with DNA. In addition to nucleic acid loading, functional groups on the surface of nanogels can be adopted so as to incorporate the nanogels with targeting ligands.

## 4. Concluding remarks

Nanogels have attracted extensive research interest for applications in targeted drug delivery, diagnosis, biosensing, and separation of biological substances (Li et al., 2002; Cheng et al., 2011; Thorne et al., 2011). Nanogels show the capacity of being loaded with diverse hydrophilic or hydrophobic agents, ranging from small-molecule compounds and proteins to nucleic acids. Upon proper design, nanogels can trigger controlled or sustained drug release; however, the residual surfactants or unreacted monomers after nanogel preparation can head to toxic effects. This is a problem that has to be solved in future research in order to enhance the translation of works from the laboratory to practical use. For those nanogels that are generated from synthetic polymers, more studies on the long-term safety profile, as well as the toxicological properties of metabolites of those polymers, should also be performed so as to confirm the safe use of the nanogels in treatment.
